# Prediction of pulmonary infection during chemotherapy in non-small cell lung cancer: development and internal validation of a nomogram

**DOI:** 10.3389/fonc.2026.1926061

**Published:** 2026-09-04

**Authors:** Jianqing Hao, Jinfeng Li, Nana Lv, Huhu Chen, Jing Huang, Yiting Han, Mei Gao, Yaya Zhang, Lingxiong Wang

**Affiliations:** 1 College of Medicine, Longdong University, Qingyang, Gansu, China; 2 Institute of Oncology, Senior Department of Oncology, The Fifth Medical Center of People's Liberation Army (PLA) General Hospital, Beijing, China

**Keywords:** chemotherapy, nomogram, non-small cell lung cancer, pulmonary infection, risk prediction model

## Abstract

**Objective:**

To develop and internally validate a nomogram for predicting pulmonary infection during chemotherapy in patients with non-small cell lung cancer (NSCLC).

**Methods:**

This retrospective study included 246 NSCLC patients receiving cytotoxic chemotherapy. The primary outcome was pulmonary infection during chemotherapy. Patients were randomly divided into training (n=196) and validation (n=50) cohorts. Predictors were selected using AIC-guided forward stepwise logistic regression to construct a nomogram. Model performance was evaluated by discrimination, calibration, threshold-based classification metrics, and decision curve analysis, with internal validation performed using bootstrap resampling.

**Results:**

Pulmonary infection occurred in 55 patients (22.4%). The final model included five predictors: absolute neutrophil count, serum albumin, age, combined immunotherapy, and chronic obstructive pulmonary disease. The apparent AUC was 0.786 (95% CI 0.703–0.869), with an optimism-corrected AUC of 0.735 and a validation AUC of 0.765 (95% CI 0.562–0.968). The nomogram classified patients into low-, intermediate-, and high-risk groups with infection rates of 5.4%, 18.2%, and 37.5%, respectively. Decision curve analysis suggested modest clinical net benefit within clinically relevant threshold probability ranges.

**Conclusion:**

This internally validated nomogram, based on routinely available pre-chemotherapy variables, may provide a practical approach for preliminary pulmonary infection risk stratification in patients with NSCLC.

## 1 Introduction

Lung cancer consistently ranks among the most prevalent malignancies globally and remains the primary driver of cancer-related mortality. Recent epidemiological estimates indicate approximately 2.48 million incident cases and 1.82 million deaths worldwide in 2022, with corresponding age-standardized rates of 23.6 and 16.8 per 100,000 population (1). Although notable reductions in male incidence and mortality have been observed in several high-income regions, the overall global disease burden persists at an alarming level, and female mortality continues to rise in numerous countries (2). Histopathologically, non-small cell lung cancer (NSCLC) constitutes the vast majority of cases, accounting for roughly 80% to 85% of all lung cancer diagnoses and representing the predominant clinical entity encountered in daily practice (3). For patients presenting with locally advanced or metastatic disease, platinum-based doublet chemotherapy—whether administered as monotherapy or in combination with immune checkpoint inhibitors—remains an indispensable component of the systemic treatment armamentarium (4).

Despite its established therapeutic efficacy, cytotoxic chemotherapy exerts profound collateral damage on the hematopoietic system and host cellular immunity, thereby markedly increasing patients’ vulnerability to infectious complications. Among these, pulmonary infection is not only the most frequently encountered but also carries the most serious clinical consequences (5, 6). The myelosuppressive effects of chemotherapeutic agents, particularly the induction of neutropenia, coupled with disruption of mucosal barrier integrity, collectively create a permissive environment for bacterial, fungal, and viral pathogens. The clinical impact is substantial: affected patients experience prolonged hospital stays, incur greater healthcare costs, and frequently face chemotherapy dose attenuation, schedule interruptions, or even outright discontinuation—all of which may compromise antitumor efficacy and adversely affect long-term survival outcomes (6–8). Consequently, the capacity to reliably identify individuals at elevated risk for pulmonary infection before initiating chemotherapy is of considerable clinical value, as it would enable the implementation of intensified surveillance and proactive preventive strategies.

The pathophysiology underlying pulmonary infection in this population is inherently multifactorial. Impaired functional status, pre-existing chronic obstructive pulmonary disease, and airway obstruction from tumor masses all contribute to defective mucociliary clearance and compromised local defense mechanisms (9). Hypoalbuminemia—frequently encountered in cancer patients—serves as a composite biomarker reflecting not only nutritional depletion but also underlying systemic inflammation and catabolic stress; its association with increased infection susceptibility and adverse outcomes has been well-documented across diverse tumor types (10). More recently, the increasing adoption of combination immunotherapy has raised additional concerns. Patients receiving checkpoint inhibitors alongside chemotherapy may face heightened infection risk, potentially mediated by the frequent use of corticosteroids for managing immune-related adverse events, as well as the inherently more advanced disease stage in this subgroup (11). These observations underscore the need for a comprehensive, multidimensional predictive framework that synthesizes both patient-related and treatment-related factors to achieve accurate risk appraisal.

In recent years, numerous studies have attempted to model the risk of pulmonary infection or other pulmonary complications in oncological settings, encompassing patients undergoing chemoradiotherapy (12), those in the postoperative period (13), and individuals receiving immunotherapy for advanced NSCLC (14). Machine learning algorithms have also been applied to predict post-chemotherapy infections. For example, Sun T et al. (15) developed a machine learning-based model for predicting lung infection after chemotherapy in lung cancer patients using multiple clinical variables and demonstrated the potential value of computational approaches in infection risk assessment.

While these efforts have contributed valuable knowledge, several methodological limitations remain. Notably, readily obtainable pre-chemotherapy parameters such as absolute neutrophil count and serum albumin have often been omitted from final models, and many published prediction tools lack rigorous internal validation with appropriate correction for overfitting, as well as comprehensive calibration reporting. Such approaches may require more complex computational infrastructure and are less readily translated into routine bedside decision-making.

To address these gaps, we undertook this retrospective investigation in NSCLC patients receiving cytotoxic chemotherapy. By utilizing easily accessible baseline clinical and laboratory indicators, we aimed to construct a parsimonious multivariable logistic regression model and an accompanying nomogram, with robust bootstrap-based internal validation and external calibration in a held-out cohort, thereby providing clinicians with a convenient tool for rapid individualized risk estimation at the point of care.

## 2 Methods

### 2.1 Study design, setting, and ethical compliance

This was a study undertaken in compliance with the Declaration of Helsinki. The study was approved by the Institutional Review Board of College of Medicine, Longdong University (Approval number: LDXY 2026-006, Date: 1 Jun 2026). Informed consent was waived because of the retrospective nature of the study, with all data anonymized for privacy protection.

This investigation was designed as a single-center, retrospective observational cohort study conducted at the Department of Medical Oncology of College of Medicine, Longdong University. We consecutively screened all NSCLC patients who initiated cytotoxic chemotherapy between January 2021 and January 2026 to minimize selection bias. The primary objective was to develop and internally validate a clinical prediction model—expressed both as a multivariable logistic regression equation and a graphical nomogram—for estimating the individual probability of pulmonary infection during the treatment period. We conducted this research in full compliance with the TRIPOD statement, applying the type 2b framework that integrates split-sample validation and bootstrap resampling for internal performance assessment. Our institutional ethics committee reviewed and approved the study protocol prior to data collection. Given the retrospective design, the exclusive use of de-identified data, and the absence of any additional patient contact or intervention, the requirement for written informed consent was formally exempted by the reviewing board.

### 2.2 Participant eligibility and exclusion criteria

The source population comprised consecutive patients with a pathologically confirmed diagnosis of NSCLC who were admitted to our ward for systemic chemotherapy. Individuals were considered eligible if they fulfilled all of the following criteria at baseline: (1) age of 18 years or older; (2) definitive histopathological or cytological diagnosis of NSCLC according to the current World Health Organization classification; and (3) administration of at least one complete cycle of intravenous cytotoxic chemotherapy (primarily platinum-based doublets, including but not limited to carboplatin or cisplatin combined with pemetrexed, paclitaxel, or gemcitabine), regardless of treatment line, with or without concurrent immune checkpoint inhibitors.

Patients were excluded from the analytical cohort if they met any of the following conditions: (1) a histologic diagnosis of small cell lung cancer or mixed histology containing small cell components, given the distinct biological behavior and treatment response of these entities; (2) the presence of clinically overt, uncontrolled active pulmonary infection documented within the 7-day interval preceding the scheduled chemotherapy start, as determined by the treating physician based on symptoms, imaging, and laboratory parameters; (3) receipt of prior thoracic radiotherapy within the preceding 3 months, which could induce radiation-induced lung changes that might confound outcome assessment; or (4) incomplete documentation for any of the prespecified candidate predictor variables or the primary outcome measure. Following this sequential screening process, 72 of the 318 initially identified patients were excluded, leaving 246 eligible patients for the final analytical sample.

### 2.3 Data extraction and measurement of candidate predictors

All clinical, demographic, and laboratory information was extracted from the institutional electronic health record system using a structured, pre-designed case report form. Baseline was operationally defined as the time point immediately preceding the first infusion of the initial chemotherapy cycle, with all measurements obtained within the 72-hour window prior to drug administration. Demographic data included age (retained as a continuous variable, in years) and sex. We used the Eastern Cooperative Oncology Group (ECOG) performance status (grades 0–4) to evaluate patients’ functional status before chemotherapy, with scores documented by the treating oncologist at the initial visit. Comorbidity data—particularly regarding COPD and diabetes—were retrieved from routine medical records. We defined COPD as a previously established physician diagnosis corroborated by pulmonary function testing showing a post-bronchodilator FEV1/FVC ratio under 0.70. For diabetes, we relied on either a known prescription of anti-diabetic agents or a fasting blood glucose concentration of 126 mg/dL or higher. Tumor stage was assigned per the eighth edition of the AJCC TNM classification, based on baseline contrast-enhanced chest and abdominal computed tomography, as well as brain imaging performed when clinically indicated.

Routine laboratory parameters were measured from peripheral blood samples drawn under standardized conditions within 7 days before the first chemotherapy cycle. The following variables were extracted from complete blood count and biochemistry panels: absolute neutrophil count (ANC, ×10^9^/L), serum albumin (g/L), C-reactive protein (CRP, mg/L), neutrophil-to-lymphocyte ratio (NLR, computed as the quotient of absolute neutrophil count divided by absolute lymphocyte count), and hemoglobin (g/L). All assays were conducted in our central clinical laboratory using automated analyzers subjected to daily internal quality control and regular external proficiency testing. Concurrent immunotherapy was defined as the simultaneous administration of a programmed death-1 (PD-1) or programmed death-ligand 1 (PD-L1) inhibitor together with the cytotoxic chemotherapy regimen during the same treatment cycle and was coded as a binary variable (0=no, 1=yes). COPD was also coded as a binary variable (0=no, 1=yes). Age, ANC, and albumin were retained as continuous predictors without categorization to preserve information and avoid arbitrary cutoff selection.

### 2.4 Outcome definition and adjudication protocol

The prespecified primary endpoint was the occurrence of pulmonary infection during the entire chemotherapy period, spanning from the day of first infusion to 30 days following the last administered cycle. To ensure diagnostic rigor, we applied a composite case definition requiring simultaneous fulfillment of four criteria: (1) new-onset or worsening respiratory symptoms (e.g., productive cough, dyspnea, pleuritic pain, or fever ≥38.0 °C); (2) radiological evidence of new or progressive infiltrates, consolidations, ground-glass opacities, or cavitary lesions on chest computed tomography or plain radiography, as interpreted by two independent radiologists; (3) laboratory or microbiological corroboration (peripheral leukocyte count ≥10.0×10^9^/L or ≤4.0×10^9^/L with a left shift, procalcitonin >0.5 ng/mL, or a positive culture from sputum, bronchoalveolar lavage, or blood); and (4) initiation of targeted antimicrobial therapy (antibacterial, antifungal, or antiviral) by the treating clinician in response to the clinical picture. Non-infectious pulmonary conditions—including radiation pneumonitis, checkpoint inhibitor-related pneumonitis, pulmonary embolism, cardiogenic edema, tumor progression with obstructive atelectasis, and drug-induced interstitial changes—were strictly excluded from the endpoint.

To minimize ascertainment bias, all potential outcome events underwent a two-tier adjudication process. Two senior investigators, fully blinded to baseline predictor values, independently reviewed each suspected case against the composite criteria. Disagreements were resolved through consensus discussion; if disagreement persisted, a third specialist in thoracic oncology and infectious diseases served as the final arbiter. The inter-rater agreement, quantified by Cohen’s kappa coefficient, was excellent at 0.89.

### 2.5 Sample size and events-per-variable consideration

We did not conduct a formal *a priori* power calculation tailored to specific effect sizes, as is customary in exploratory prediction modeling research. Instead, we evaluated the feasibility of the planned model by examining the events-per-variable (EPV) ratio. With 55 outcome events observed in the full cohort (246 patients), the overall events-per-variable (EPV) ratio was approximately 5.5 based on 10 candidate predictors. After random allocation, the training cohort included 196 patients with 47 pulmonary infection events, corresponding to an EPV of approximately 4.7, whereas the independent validation cohort consisted of 50 patients with 8 events. Although the EPV was below the traditional recommendation of 10 events per variable, this study was designed as an exploratory prediction model development study, and several methodological strategies were implemented to mitigate the potential risk of overfitting. To counter the attendant risk of overfitting and coefficient instability, we implemented several pre-specified protective measures, including restriction of candidate predictors according to clinical relevance, penalized model complexity through AIC-guided forward selection rather than simple P-value-based selection, and bootstrap-based internal validation incorporating the entire variable selection process. (1) restriction of the candidate predictor pool to clinically and biologically relevant variables only, avoiding exhaustive inclusion of all available covariates; (2) utilization of AIC-driven stepwise selection, which imposes a more stringent penalty for model complexity compared to P-value-based selection; and (3) application of a rigorous bootstrap internal validation scheme that fully replicates the variable selection process in each resample, thereby providing a realistic estimate of optimism.

### 2.6 Data splitting and model building process

The complete-case dataset was randomly divided into a training cohort (n=196) and a validation cohort (n=50) using an 8:2 allocation ratio. Randomization was executed with a fixed pseudo-random seed to guarantee complete reproducibility. The training cohort was dedicated exclusively to variable selection, model fitting, and nomogram construction; the validation cohort was preserved strictly for final calibration appraisal and was not accessed during any phase of model development. Because pulmonary infection occurred in only 55 of 246 patients (22.4%), the dataset showed moderate class imbalance. We did not apply artificial resampling approaches, such as the Synthetic Minority Oversampling Technique (SMOTE), during model development, because altering the event prevalence may distort predicted probabilities and compromise calibration accuracy. Instead, we retained the original event distribution and addressed potential imbalance-related limitations through comprehensive performance evaluation, including calibration assessment, Brier score calculation, decision curve analysis, and threshold-based classification metrics.

In the training cohort, candidate predictors were selected based on a combination of clinical biological plausibility and data-driven statistical optimization. Variables were prespecified according to their established relationships with chemotherapy-related infectious complications, including hematopoietic suppression (absolute neutrophil count), nutritional and inflammatory status (serum albumin), host vulnerability (age and COPD), and treatment-related immune modulation (combined immunotherapy). Considering the limited number of outcome events and the potential risk of overfitting, we intentionally restricted the candidate predictor pool and avoided excessive inclusion of correlated variables. Variables attaining a univariable P-value <0.20 were considered for entry into the multivariable model; however, the final selection was determined by the Akaike information criterion using a Forward-AIC strategy rather than simple statistical significance.

Utilizing a forward stepwise algorithm with AIC as the selection metric, we sequentially incorporated predictors into an intercept-only model. At each step, the candidate variable producing the largest reduction in AIC was entered. The procedure continued until no remaining candidate variable produced further AIC reduction. After each addition, previously entered variables were reassessed and removed if their exclusion resulted in a lower AIC, ensuring selection of the most parsimonious model. No hyperparameter tuning or automated machine-learning optimization was performed because the objective was development of an interpretable clinical prediction model.

At each iterative step, we also evaluated the potential removal of previously entered variables to ensure the final model represented the most parsimonious yet adequately informative set. All continuous predictors were analyzed on their original scale without arbitrary categorization, as dichotomization is known to sacrifice information and statistical power. We further examined multicollinearity among the selected variables using variance inflation factor (VIF) calculations; all VIF values were below 2.0, effectively excluding problematic collinearity.

### 2.7 Nomogram development and scoring scheme

Based on the final multivariable logistic regression coefficients, we constructed a high-resolution nomogram to translate the mathematical model into a practical clinical scoring system. For each predictor, a point value was derived by multiplying the regression coefficient by a constant factor and rounding to the nearest integer. Reference values for continuous variables were anchored at clinically meaningful thresholds (e.g., medians or normal lower limits) to define baseline points. Individual scores were summed to yield a total score, which was then transformed via the logistic function—Probability = 1/(1 + exp(−(β_0_ + Σβ_i_X_i_)))—to generate the predicted probability of pulmonary infection. This graphical tool was designed to enable rapid visual estimation of risk without requiring electronic computation, thus facilitating seamless integration into routine bedside practice.

### 2.8 Performance evaluation metrics

Model performance was comprehensively assessed across four complementary dimensions: discrimination, calibration, classification accuracy, and clinical utility.

Model discrimination, defined as the ability to correctly differentiate between patients who developed pulmonary infection and those who remained free of it, was evaluated using the area under the receiver operating characteristic curve (AUC). Considering the relatively low event prevalence, threshold-dependent classification metrics were additionally calculated to provide clinically interpretable measures of performance. At the predefined high-risk threshold (predicted probability ≥20%, corresponding to the clinically relevant intervention cutoff), we calculated sensitivity (recall), specificity, precision, positive predictive value (PPV), negative predictive value (NPV), and Matthews correlation coefficient (MCC). These metrics were reported together with AUC to avoid overreliance on discrimination measures alone. In this metric, a value of 0.5 denotes performance no better than random assignment, whereas a value of 1.0 reflects flawless discriminative power. We also computed the Brier score, an overall accuracy metric that integrates both discrimination and calibration; lower values (closer to 0) indicate superior predictive performance, with a reference benchmark of 0.25 representing the variance of a binary outcome with 50% prevalence.

To examine calibration, i.e., how closely the predicted risks matched the observed infection frequencies, we plotted calibration curves separately for the training and validation samples. For the training set, we further conducted the Hosmer-Lemeshow test; a P-value exceeding 0.05 indicated that the model’s predicted probabilities did not deviate significantly from the actual event rates, supporting adequate calibration. In the validation cohort, where sample size limited the power of formal testing, we estimated the calibration intercept (indicating overall systematic over- or under-estimation) and the calibration slope (reflecting the average effect magnitude of the linear predictor); an intercept near 0 and a slope close to 1 denote perfect calibration. The expected-to-observed (E/O) ratio was additionally calculated as a supplementary calibration metric.

Clinical utility was appraised using decision curve analysis (DCA), which quantifies the net benefit associated with using the prediction model across a spectrum of clinically sensible threshold probabilities for intervention. Unlike purely statistical performance measures, DCA incorporates the relative clinical consequences of false-positive and false-negative decisions, thereby offering a patient-centered perspective on whether the model adds meaningful value relative to default strategies of treating all or treating none.

### 2.9 Internal validation and optimism correction

To account for the unavoidable overfitting inherent in model development, we performed internal validation using bootstrap resampling with 1,000 iterations to obtain a more stable estimation of optimism. In each bootstrap replicate, the entire modeling pipeline was repeated, including candidate variable screening, Forward-AIC-based variable selection, model refitting, and coefficient estimation. This procedure allowed optimism correction to account not only for coefficient shrinkage but also for uncertainty related to predictor selection. The apparent AUC was computed for each bootstrap sample (bootstrap performance), and the identical model was subsequently applied to the original training cohort (test performance). The optimism for each replicate was calculated as the difference between bootstrap and test performance; the mean optimism across all 1,000 bootstrap replicates was then subtracted from the original apparent AUC to obtain the optimism-corrected AUC. This approach furnishes a more honest estimation of expected performance in future patient populations, as it penalizes for uncertainty in both predictor selection and coefficient shrinkage. Additionally, we recorded the selection frequency of each variable across the 1,000 bootstrap replicates as an index of variable stability, providing an assessment of the robustness of predictor selection under repeated sampling.

### 2.10 Risk stratification and exploratory dynamic prediction

To enhance clinical interpretability, we stratified patients in the training cohort into three distinct risk categories according to their predicted probabilities: low (<10%), intermediate (10%–20%), and high (≥20%). These cutoff values were selected based on clinical consensus regarding actionable intervention thresholds; we also performed sensitivity analyses using alternative boundaries (e.g., 15% and 25%) to test the robustness of stratification.

Beyond the static patient-level model, we conducted an exploratory landmark-based dynamic prediction analysis at the chemotherapy-cycle level. For each treatment cycle (cycles 1 through 4, where sufficient numbers of at-risk patients remained), we defined a landmark time point at cycle initiation. Among patients who remained infection-free at that landmark, we applied the baseline predictor set (identical to the primary model) to estimate the probability of infection during the subsequent cycle. Performance for each prediction window (cycle 1→2, 2→3, and 3→4) was evaluated using the AUC, and corresponding next-cycle infection rates were calculated. While this exploratory analysis used time-fixed baseline covariates rather than time-updated measurements, it was designed only to evaluate whether the baseline model retained discriminative ability across successive chemotherapy cycles. Dynamic updating based on longitudinal laboratory parameters was not incorporated because repeated measurements were unavailable for all patients.

### 2.11 Missing data and sensitivity analyses

The primary analysis was restricted to complete cases, as patients with missing data for key predictors or the outcome were excluded per our pre-specified criteria. To evaluate whether this exclusion introduced meaningful selection bias, we conducted a *post-hoc* sensitivity comparison of baseline characteristics between included and excluded patients; no statistically significant differences emerged in terms of age, sex, or major comorbidities, suggesting that the missingness mechanism was likely at random. Missing data were evaluated according to their distribution patterns and potential mechanisms. Although only 12 patients were excluded because of incomplete predictor or outcome information, we performed multiple imputation sensitivity analyses to assess the robustness of the complete-case findings. Missing values were imputed using multiple imputation by chained equations (MICE), generating 20 imputed datasets under the missing-at-random assumption. The prediction model was reconstructed in each imputed dataset, and pooled estimates were obtained according to Rubin’s rules. The results were compared with the primary complete-case analysis regarding regression coefficients, odds ratios, and model discrimination.

### 2.12 Software and reproducibility

All statistical analyses were conducted using Python version 3.11. The statsmodels library (version 0.14.0) was employed for logistic regression and associated diagnostic procedures; scipy (version 1.11.0) was used for non-parametric tests and normal approximation methods; and scikit-learn (version 1.3.0) was utilized for AUC calculation, bootstrap implementation, and calibration curve generation. Decision curve analysis was performed using a custom script implementing the standard net benefit formulation, validated against published R-based implementations. The complete analysis code, including data-splitting procedures and bootstrap routines with fixed random seeds, is available from the corresponding author upon reasonable request to ensure full transparency and reproducibility.

### 2.13 Statistical reporting conventions

All hypothesis tests were two-tailed, and a P-value <0.05 was considered to denote statistical significance. Continuous data were reported as medians with interquartile ranges (IQR), while categorical data were summarized as absolute frequencies with percentages. Regression outputs were presented as odds ratios with accompanying 95% confidence intervals. Confidence intervals for AUCs were estimated using the Hanley-McNeil method, which provides a closed-form approximation based on the standard error of the AUC. All figures and tables were generated following principles of clear and concise graphical presentation to facilitate interpretation for clinical readers.

## 3 Results

### 3.1 Baseline characteristics of the study population

**Figure 1:**
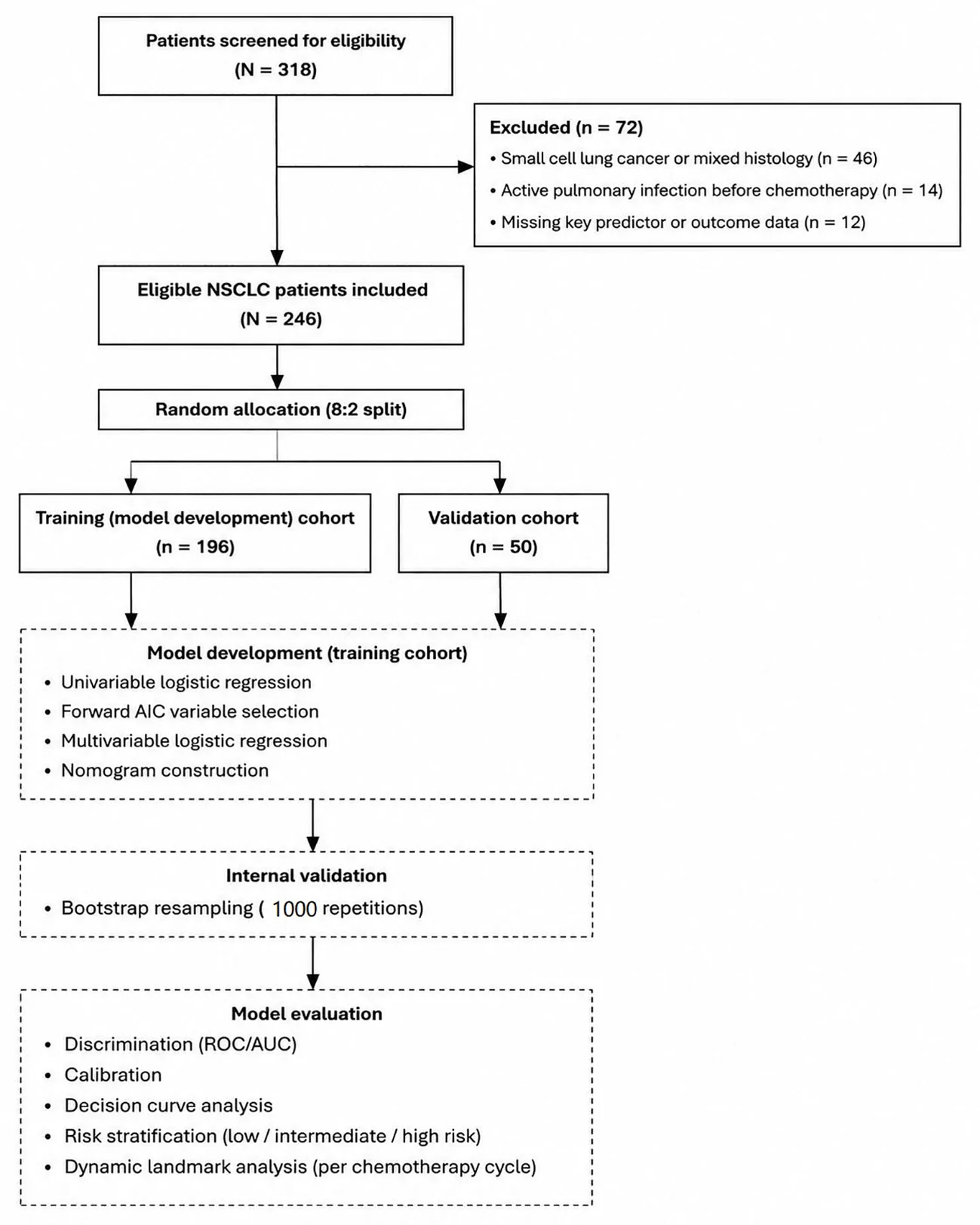
Flow diagram of patient selection and cohort allocation.

Over the study enrollment period, 318 lung cancer patients receiving cytotoxic chemotherapy were initially screened. After applying the exclusion criteria, 72 patients were excluded, including 46 with small cell histology or mixed small-cell components, 14 with uncontrolled active pulmonary infection before chemotherapy, and 12 with incomplete documentation of key variables or outcome information. Therefore, 246 patients with NSCLC were included in the final analytic cohort (**Figure 1**).

During the chemotherapy course, pulmonary infection occurred in 55 patients (22.4%). The median age of the cohort was 62.3 years (IQR 55.0–67.6), and 154 patients (62.6%) were male. ECOG performance status was 0 in 59 patients (24.0%), 1 in 114 (46.3%), 2 in 60 (24.4%), and 3 in 13 (5.3%). Most patients had advanced disease, with 94 (38.2%) classified as stage III and 112 (45.5%) as stage IV. COPD and diabetes mellitus were present in 58 (23.6%) and 45 patients (18.3%), respectively. Concurrent immunotherapy was administered to 73 patients (29.7%).

An exploratory analysis of corticosteroid exposure among patients receiving combined immunotherapy showed that 18 patients (24.7%) received corticosteroids during chemotherapy, mainly for the management of immune-related adverse events. Corticosteroid exposure was observed more frequently among patients who developed pulmonary infection than among those without infection (37.5% vs. 18.4%), although this difference did not reach statistical significance (P = 0.098).

Compared with patients without infection, those who developed pulmonary infection had significantly lower baseline ANC [3.3 (2.2–3.8) vs. 4.2 (3.2–5.1) ×10^9^/L, P<0.001] and serum albumin levels [35.1 (33.0–38.9) vs. 37.8 (34.8–41.3) g/L, P = 0.005]. Concurrent immunotherapy was more common among infected patients (43.6% vs. 25.7%, P = 0.016). Age and COPD prevalence showed increasing trends in the infection group but did not reach statistical significance (**Table 1**).

**Table 1:** Baseline characteristics of the cohort.

Characteristic	Total (N = 246)	No infection (n=191)	Infection (n=55)	P value
Age, years	62.3 (55.0–67.6)	61.3 (54.6–67.1)	64.6 (57.2–68.2)	0.089
Male sex	154 (62.6)	121 (63.4)	33 (60.0)	0.768
ECOG performance status				0.108
0	59 (24.0)	45 (23.6)	14 (25.5)	
1	114 (46.3)	94 (49.2)	20 (36.4)	
2	60 (24.4)	45 (23.6)	15 (27.3)	
3	13 (5.3)	7 (3.7)	6 (10.9)	
TNM stage				0.916
I	17 (6.9)	14 (7.3)	3 (5.5)	
II	23 (9.3)	17 (8.9)	6 (10.9)	
III	94 (38.2)	74 (38.7)	20 (36.4)	
IV	112 (45.5)	86 (45.0)	26 (47.3)	
COPD	58 (23.6)	40 (20.9)	18 (32.7)	0.102
Diabetes mellitus	45 (18.3)	32 (16.8)	13 (23.6)	0.334
Combined immunotherapy	73 (29.7)	49 (25.7)	24 (43.6)	0.016
ANC, ×10^9^/L	3.9 (2.9–4.9)	4.2 (3.2–5.1)	3.3 (2.2–3.8)	<0.001
Albumin, g/L	37.1 (34.3–40.6)	37.8 (34.8–41.3)	35.1 (33.0–38.9)	0.005
CRP, mg/L	9.2 (4.6–17.5)	9.1 (4.7–16.5)	10.6 (3.6–21.1)	0.527
NLR	3.4 (2.5–4.6)	3.3 (2.5–4.5)	3.8 (2.5–4.7)	0.309
Hemoglobin, g/L	120.2 (108.2–132.2)	120.3 (108.3–132.0)	119.9 (107.8–133.9)	0.986
Chemotherapy cycle				0.110
1	27 (11.0)	25 (13.1)	2 (3.6)	
2	46 (18.7)	32 (16.8)	14 (25.5)	
3	56 (22.8)	46 (24.1)	10 (18.2)	
4	69 (28.0)	50 (26.2)	19 (34.5)	
5	37 (15.0)	31 (16.2)	6 (10.9)	
6	11 (4.5)	7 (3.7)	4 (7.3)	

Data are presented as median (interquartile range) or number of cases (%). The Mann-Whitney U test was used for continuous variables, and the chi-square test was used for categorical variables. ANC, absolute neutrophil count; CRP, C-reactive protein; NLR, neutrophil-to-lymphocyte ratio; ECOG, Eastern Cooperative Oncology Group; COPD, chronic obstructive pulmonary disease.

### 3.2 Factors associated with pulmonary infection in regression analyses

In the univariable logistic regression analyses, age, ANC, albumin, ECOG score, and concurrent immunotherapy were associated with pulmonary infection. Although age showed only a borderline difference in baseline group comparison (P = 0.089), univariable regression demonstrated a significant association with infection risk (OR 1.04, 95% CI 1.00–1.08, P = 0.030). After adjustment for other clinically important predictors, age remained independently associated with pulmonary infection (OR 1.05, 95% CI 1.01–1.10, P = 0.016). After adjustment for other clinically important predictors, including ANC and albumin, age became independently associated with pulmonary infection (OR 1.05, 95% CI 1.01–1.10, P = 0.016), suggesting a potential confounding or suppression effect. CRP, NLR, hemoglobin, cycle number, COPD, and diabetes did not achieve statistical significance.

The forward AIC-based stepwise selection procedure retained five variables in the final multivariable model.

The final logistic regression equation was:


$$\text{logit}\left(\text{P}\right)=\beta_{0}\;+\;\beta_{1}\left(\text{ANC}\right)\;+\;\beta_{2}\left(\text{Albumin}\right)\;+\;\beta_{3}\left(\text{Age}\right)\;+\;\beta_{4}\left(\text{Immunotherapy}\right)\;+\;\beta_{5}\left(\text{COPD}\right)$$

where:


logit(P)=ln[P/(1−P)].

The estimated coefficients were β_0_=1.842, βANC=−0.544 (SE = 0.142), βAlbumin=−0.083 (SE = 0.039), βAge=0.049 (SE = 0.020), βImmunotherapy=0.881 (SE = 0.387), and βCOPD=0.724 (SE = 0.414).

Continuous predictors were entered as original continuous values. Immunotherapy and COPD were binary variables coded as 1=yes and 0=no (**Supplementary Table 1**).

**Figure 2:**
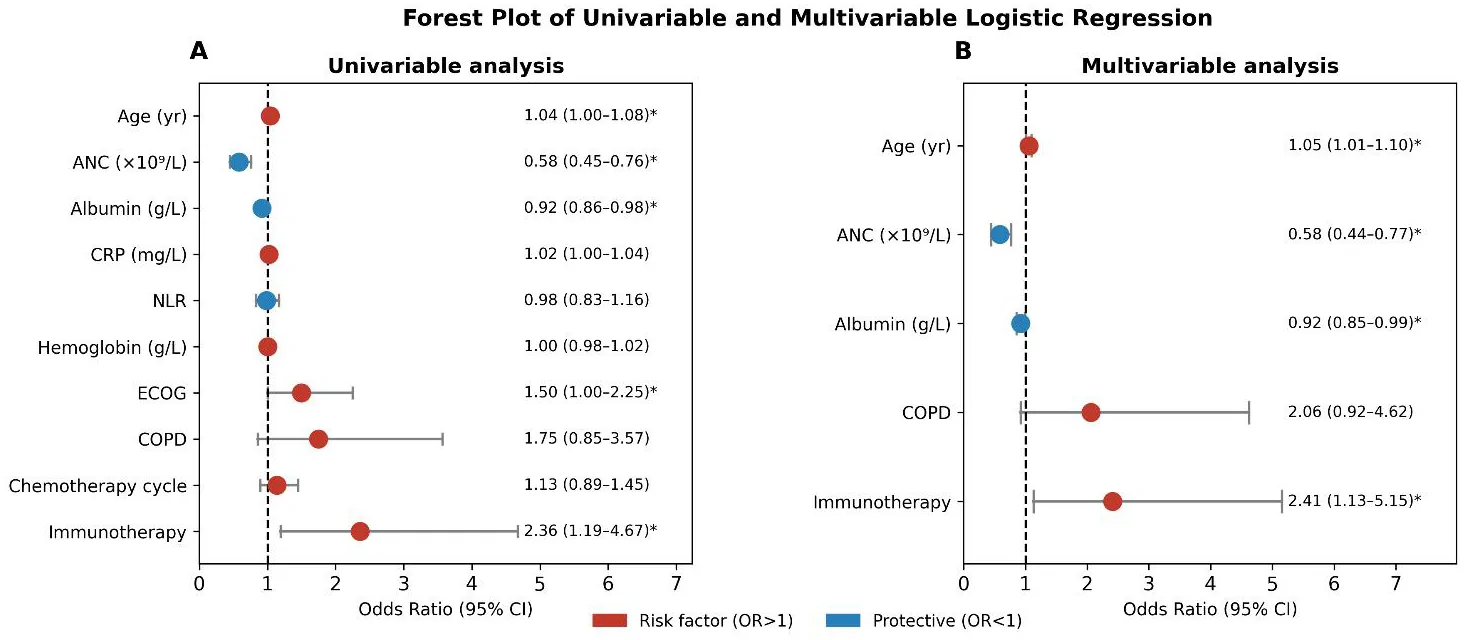
Forest plot of univariable and multivariable logistic regression analyses for pulmonary infection risk. **(A)** Univariable and **(B)** multivariable logistic regression analyses. Dots represent odds ratios (OR) and horizontal lines the 95% confidence intervals (CI); the vertical dashed line marks OR = 1. Red denotes risk factors (OR > 1) and blue protective factors (OR < 1). *P < 0.05.

Higher ANC maintained its protective effect (OR 0.58, 95% CI 0.44–0.77, P<0.001), and elevated albumin also remained protective (OR 0.92, 95% CI 0.85–0.99, P = 0.035). Increasing age (OR 1.05, 95% CI 1.01–1.10, P = 0.016) and concurrent immunotherapy (OR 2.41, 95% CI 1.13–5.15, P = 0.023) emerged as independent risk factors. COPD demonstrated a risk-increasing tendency, though it did not attain formal statistical significance (**Table 2**; **Figure 2**).

**Table 2:** Univariable and multivariable logistic regression.

Variable	Crude OR (95% CI)	P value	Adjusted OR (95% CI)	P value
Age, per year	1.04 (1.00–1.08)	0.030	1.05 (1.01–1.10)	0.016
ANC, per ×10^9^/L	0.58 (0.45–0.76)	<0.001	0.58 (0.44–0.77)	<0.001
Albumin, per g/L	0.92 (0.86–0.98)	0.015	0.92 (0.85–0.99)	0.035
CRP, per mg/L	1.02 (1.00–1.04)	0.109	—	—
NLR, per unit	0.98 (0.83–1.16)	0.850	—	—
Hemoglobin, per g/L	1.00 (0.98–1.02)	0.842	—	—
ECOG, per grade	1.50 (1.00–2.25)	0.049	—	—
COPD (yes vs no)	1.75 (0.85–3.57)	0.126	2.06 (0.92–4.62)	0.079
Chemotherapy cycle, per cycle	1.13 (0.89–1.45)	0.308	—	—
Immunotherapy (yes vs no)	2.36 (1.19–4.67)	0.014	2.41 (1.13–5.15)	0.023

Based on the modeling cohort (training set, n=196). Variables in the multivariate model were selected via the Forward-AIC stepwise method. OR, odds ratio; CI, confidence interval; “—” indicates the variable was not included in the multivariate model.

### 3.3 Construction of the nomogram

**Figure 3:**
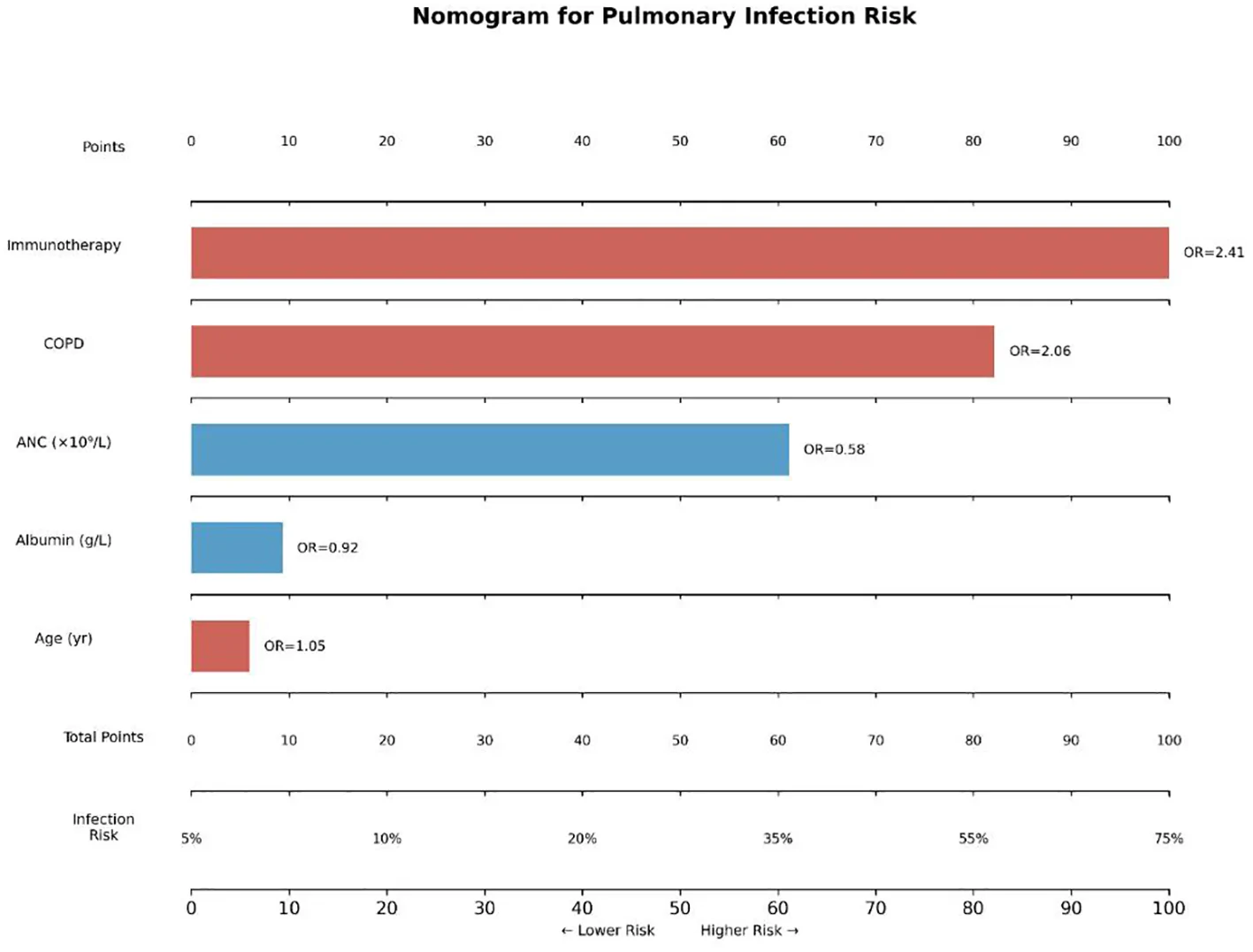
Risk prediction nomogram based on five independent predictors for pulmonary infection during chemotherapy in patients with non-small cell lung cancer (NSCLC). The nomogram was developed based on five independent predictors identified in the multivariable logistic regression model, including absolute neutrophil count (ANC), serum albumin, age, concurrent immunotherapy, and chronic obstructive pulmonary disease (COPD). Example interpretation: A 65-year-old patient with ANC of 3.0×10^9^/L, serum albumin of 35 g/L, concurrent immunotherapy, and COPD would be classified as having a relatively high predicted risk, corresponding to an estimated probability of pulmonary infection of approximately 35%–40%. This example demonstrates the application of the nomogram for individualized risk estimation before chemotherapy initiation.

A high-resolution nomogram was developed based on the regression coefficients from the final multivariable model (**Figure 3**). The nomogram incorporated five independent predictors: ANC, serum albumin, age, concurrent immunotherapy, and COPD. For practical application, an example case was provided. A 65-year-old NSCLC patient scheduled for chemotherapy with ANC of 3.0×10^9^/L, serum albumin of 35 g/L, concurrent immunotherapy (yes), and COPD (yes) would be classified as having a relatively high predicted risk, corresponding to an estimated pulmonary infection probability of approximately 35%–40%. This example illustrates the potential utility of the nomogram for individualized risk estimation before chemotherapy initiation.

The contribution of each predictor was determined according to its regression coefficient. Higher ANC and albumin levels contributed toward lower predicted risk, reflecting their protective effects, whereas older age, concurrent immunotherapy, and COPD increased the estimated probability of pulmonary infection. The nomogram therefore provides an intuitive approach for bedside risk stratification.

### 3.4 Model discrimination, calibration, and internal validation

**Figure 4:**
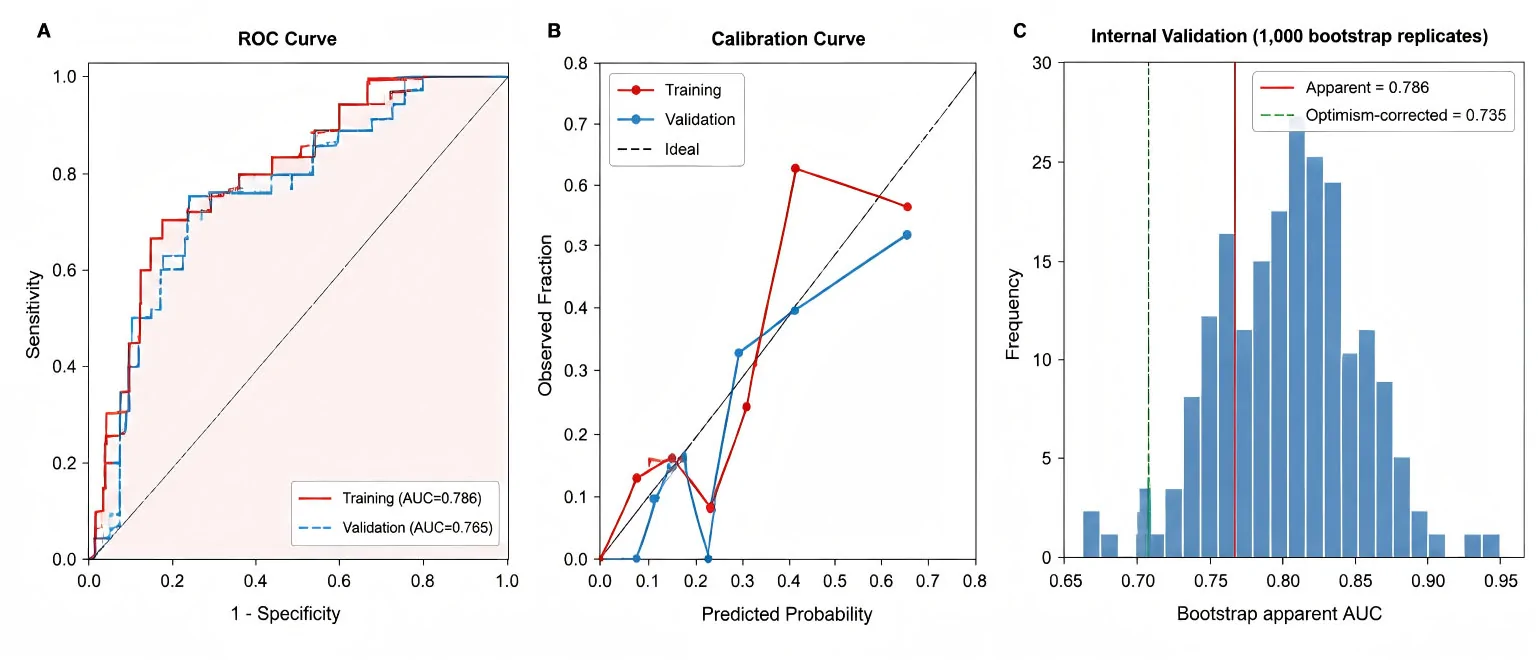
Performance evaluation of the prediction model. **(A)** Receiver operating characteristic (ROC) curves demonstrating model discrimination. The area under the curve (AUC) was 0.786 (95% CI 0.703–0.869) in the training cohort and 0.765 (95% CI 0.562–0.968) in the validation cohort. **(B)** Calibration curves showing the agreement between predicted and observed risks in the training and validation cohorts. The diagonal dashed line represents perfect calibration; in the validation cohort, the calibration intercept was −0.555 and the calibration slope was 0.850. **(C)** Internal validation using 1,000 bootstrap replicates with repeated variable selection and model refitting procedures. The apparent AUC was 0.786, and the optimism-corrected AUC was 0.735, corresponding to an estimated optimism of 0.051.

In the training cohort, the model exhibited an apparent AUC of 0.786 (95% CI 0.703–0.869) and a Brier score of 0.148; in the validation cohort, the corresponding values were 0.765 (95% CI 0.562–0.968) and 0.129, respectively, indicating consistent discriminatory performance (**Figure 4A**). Calibration assessment showed acceptable agreement between predicted and observed risks in both cohorts (**Figure 4B**). In the validation cohort, the calibration intercept was −0.555 and the slope was 0.850, suggesting slight overestimation of predicted risk, particularly at higher probability ranges; however, these estimates should be interpreted cautiously given the limited validation sample size. Through bootstrap resampling with 1,000 replicates, including repeated variable selection, model refitting, and coefficient estimation procedures, the estimated optimism was 0.051 (95% percentile interval: 0.018–0.084), yielding an optimism-corrected AUC of 0.735 (**Figure 4C**). These findings indicate moderate optimism due to the limited event number but support the internal stability of the model.

Performance metrics and validation outcomes are summarized in **Table 3** and **Figure 4**. Given the imbalance between infection and non-infection cases, additional threshold-based classification analyses were performed. At the clinically predefined high-risk threshold of ≥20%, the model achieved a sensitivity of 81.8%, precision of 37.5%, PPV of 37.5%, NPV of 92.0%, and MCC of 0.39 in the training cohort (**Table 3**).

**Table 3:** Model discrimination, calibration, and threshold-based classification performance.

Cohort/performance assessment	AUC (95% CI)	Brier score	Threshold	TP	FP	TN	FN	Sensitivity (Recall)	Specificity	Precision	PPV	NPV	MCC
Training set (n=196), apparent	0.786 (0.703–0.869)	0.148	≥20% predicted risk	38	59	90	9	80.90%	60.40%	39.20%	39.20%	90.90%	0.39
Optimism-corrected (bootstrap)	0.735	—	—	—	—	—	—	—	—	—	—	—	—
Validation set (n=50)	0.765 (0.562–0.968)	0.129	≥20% predicted risk	6	16	26	2	75.00%	61.90%	27.30%	27.30%	92.90%	0.32

AUC 95%CI: Estimated using the Hanley-McNeil method; Internal validation adopted bootstrap resampling with 1,000 replicates, with the complete variable selection and model fitting procedures repeated in each replicate, to estimate optimism bias (=0.051) and obtain the optimism-corrected AUC. Calibration was assessed in the validation cohort (intercept −0.555, slope 0.850). AUC, area under the curve.

Sensitivity analyses using multiple imputation yielded highly consistent results with the primary complete-case analysis. In the pooled imputed datasets, the five predictors retained the same direction of association, including protective effects of ANC (OR 0.60, 95% CI 0.46–0.79) and albumin (OR 0.93, 95% CI 0.86–0.99), and increased risks associated with age (OR 1.05, 95% CI 1.01–1.10), combined immunotherapy (OR 2.34, 95% CI 1.10–4.98), and COPD (OR 2.01, 95% CI 0.90–4.49). The pooled AUC was 0.781, which was comparable with the complete-case estimate (AUC = 0.786), indicating that exclusion of patients with incomplete data did not materially influence model performance.

### 3.5 Risk stratification, clinical utility, and dynamic prediction

**Figure 5:**
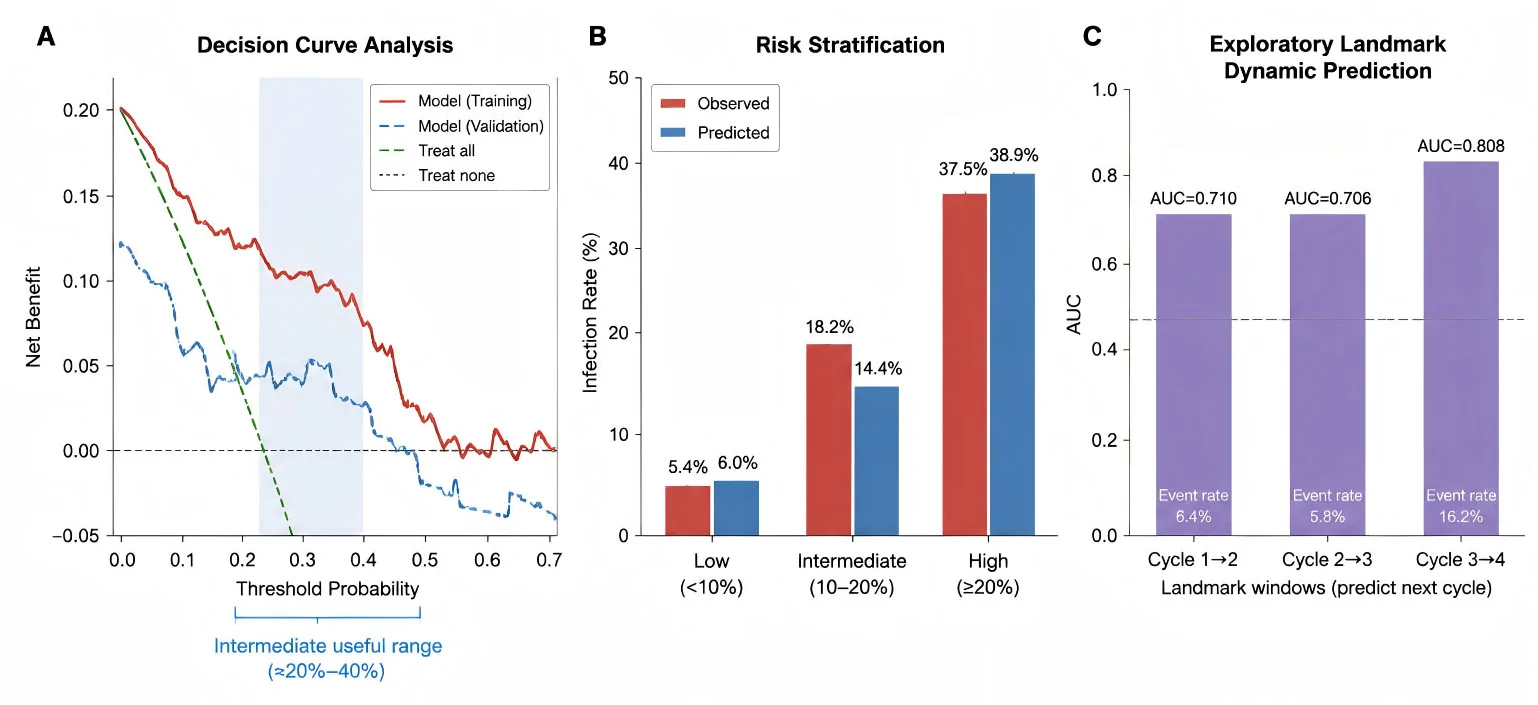
Clinical application and exploratory dynamic assessment of the prediction model. **(A)** Decision curve analysis demonstrating the clinical net benefit of the prediction model compared with “treat-all” and “treat-none” strategies. The model showed modest incremental net benefit mainly within an intermediate threshold probability range of approximately 20%–40%, while the overall decision utility should be interpreted cautiously given the limited event rate and validation sample size. **(B)** Risk stratification according to predicted probabilities. Patients were classified into low-, intermediate-, and high-risk groups, with observed infection rates increasing progressively across strata. **(C)** Exploratory landmark dynamic prediction analysis across chemotherapy cycles. The model maintained acceptable discrimination for predicting subsequent-cycle pulmonary infection, with AUC values of 0.710, 0.706, and 0.808 for cycle 1→2, cycle 2→3, and cycle 3→4 prediction windows, respectively.

Based on the predicted probabilities, patients in the training cohort were classified into low-, intermediate-, and high-risk strata. Decision curve analysis demonstrated that the model provided incremental net benefit compared with the “treat-all” and “treat-none” strategies mainly within an intermediate threshold probability range (approximately 20%–40%), although the absolute magnitude of net benefit was modest (**Figure 5A**). The observed infection rates showed a consistent increasing gradient across risk groups and were generally comparable with the corresponding mean predicted risks: 5.4% versus 6.0% in the low-risk group, 18.2% versus 14.4% in the intermediate-risk group, and 37.5% versus 38.9% in the high-risk group (**Table 4A**; **Figure 5B**). Although the absolute net benefit was modest and the clinically useful threshold range was relatively narrow, this range overlaps with risk levels at which intensified surveillance or preventive strategies may be considered for selected high-risk patients. Therefore, the nomogram should be interpreted primarily as a risk stratification tool rather than a definitive clinical decision rule. Therefore, the model should be interpreted as a risk stratification tool rather than a definitive treatment decision rule.

**Table 4:** Risk stratification and landmark dynamic prediction.

A. Risk stratification by predicted probability				
Risk group	Patients, n	Events, n	Observed rate, %	Mean predicted, %
Low (<10%)	56	3	5.4	6.0
Intermediate (10–20%)	44	8	18.2	14.4
High (≥20%)	96	36	37.5	38.9

B. Landmark dynamic prediction				
Landmark window	At-risk cycles, n	Next-cycle infection, %		AUC
Cycle 1 → 2	219	6.4		0.710
Cycle 2 → 3	173	5.8		0.706
Cycle 3 → 4	117	16.2		0.808

(A) Stratified thresholds: low risk <10%, moderate risk 10–20%, high risk ≥20% (modeling cohort). (B) Each chemotherapy cycle is taken as a landmark time point to predict the risk of pulmonary infection in the next cycle; only patients without infection in the current cycle are included.

To explore temporal changes in risk, an exploratory landmark dynamic prediction analysis was performed (**Table 4B**; **Figure 5C**). The AUCs for predicting infection during the subsequent cycle were 0.710, 0.706, and 0.808 for cycle 1→2, cycle 2→3, and cycle 3→4, respectively, with corresponding infection rates of 6.4%, 5.8%, and 16.2%. These findings suggest that the model retained acceptable discriminative performance across successive chemotherapy cycles, with improved discrimination observed during later cycles when infection incidence increased.

## 4 Discussion

In this retrospective investigation, we developed and preliminarily validated a multivariable logistic regression model and an accompanying nomogram for predicting pulmonary infection during chemotherapy in 246 patients with NSCLC. The final model included five readily accessible pre-chemotherapy indicators: absolute neutrophil count, serum albumin, age, concurrent immunotherapy, and COPD. The apparent AUC was 0.786, and after bootstrap optimism correction that fully accounted for the variable selection process, the corrected AUC was 0.735. The nomogram stratified patients into three distinct risk tiers with progressively increasing infection rates, offering a clinically accessible tool for early identification of high-risk individuals before chemotherapy initiation.

The selection of these five predictors was guided by both biological rationale and statistical parsimony. ANC reflects the core component of host antibacterial defense and directly captures chemotherapy induced myelosuppression, a well established determinant of infection susceptibility. Serum albumin serves as an integrated marker of nutritional reserve, systemic inflammation, and overall disease burden. Age and COPD represent host vulnerability factors associated with impaired immune function and reduced pulmonary defense capacity. Concurrent immunotherapy was included as a treatment related factor that reflects the evolving therapeutic landscape of advanced NSCLC. Given the limited number of outcome events, we intentionally restricted the final model to a small set of clinically meaningful variables using Forward-AIC selection to balance predictive performance against model stability.

Among all predictors, ANC emerged as the most robust protective factor, with a selection frequency of 98.5% across bootstrap resamples. This finding aligns closely with the core pathophysiology of chemotherapy related infectious complications, in which infection risk is intimately linked to both the depth and duration of neutropenia, as lower neutrophil counts severely compromise pathogen clearance (16). Although severe, protracted neutropenia occurs less frequently in patients with solid tumors than in those with hematological malignancies, the former group often carries multiple concurrent risk factors, making the overall infectious burden clinically substantial (17). Febrile neutropenia, in particular, has been associated with markedly elevated mortality rates (7). Serum albumin showed a protective effect in the opposite direction. Hypoalbuminemia is not merely a marker of nutritional deficiency; it also reflects systemic inflammation and catabolic stress, which are common in advanced cancer (18). Its consistent association with infection susceptibility and poor prognosis across various tumor types (19) lends biological plausibility to its predictive role in this setting.

Of particular clinical relevance is the independent risk effect of concurrent immunotherapy (OR 2.41). Because our outcome definition explicitly excluded checkpoint inhibitor related pneumonitis, this association should not be interpreted as a direct causal consequence of immune checkpoint inhibition. Rather, it likely arises from indirect mechanisms and residual confounding. Patients receiving chemo-immunotherapy generally have more advanced disease, higher tumor burden, and poorer baseline immune function. In addition, management of immune related adverse events frequently requires systemic corticosteroid therapy, a well established risk factor for opportunistic infections (20, 21). In our exploratory analysis, corticosteroid use was more common among infected patients receiving immunotherapy than among those without infection (37.5% vs. 18.4%), although the difference did not reach statistical significance, possibly due to the small subgroup sample size. Previous comparative studies have similarly reported higher infection rates with chemo-immunotherapy combinations than with chemotherapy alone (22). These observations suggest that patients receiving combined immunotherapy warrant closer infectious surveillance, yet the association should be viewed as predictive rather than causal, given that corticosteroid exposure, disease severity, and treatment selection may all contribute to the observed relationship.

Age demonstrated an interesting confounding or suppression effect in our analysis. The univariable comparison showed only a borderline difference between groups (P = 0.089), whereas multivariable adjustment for ANC, albumin, and other covariates revealed an independent association (OR 1.05, P = 0.016). This pattern suggests that younger patients with severe disease related immune impairment or unfavorable treatment related factors may have partially masked the contribution of aging in the crude analysis, and that multivariable adjustment allowed the independent effect of age related vulnerability to become apparent. Advancing age, along with COPD, showed risk increasing trends, consistent with the biological phenomena of immunosenescence and impaired airway defense mechanisms resulting from structural damage and mucociliary dysfunction. The detrimental prognostic impact of COPD in NSCLC patients receiving chemotherapy has been previously documented (23, 24).

Compared with previous prediction models for pulmonary infection, our study has several methodological strengths. Sun T et al. (15) recently developed a machine learning based model for predicting lung infection after chemotherapy in lung cancer patients, demonstrating the promise of advanced computational algorithms in this field. However, machine learning approaches typically require larger datasets, specialized analytical pipelines, and external validation before broad clinical deployment (25). Our study adhered to the TRIPOD reporting framework for prediction model development and validation (26), providing a transparent logistic regression based nomogram using five routinely available variables that enables direct bedside risk estimation without additional computational resources. We also performed bootstrap based optimism correction with repeated variable selection procedures, yielding a more conservative and realistic assessment of model performance.

From a clinical decision making perspective, the DCA findings should be interpreted with caution. The net benefit was not uniformly high across all threshold probabilities, reflecting the relatively low incidence of pulmonary infection and the potential burden of unnecessary interventions. Nonetheless, within an intermediate threshold probability range of approximately 20% to 40%, the nomogram showed some incremental clinical value compared with universal monitoring or no targeted intervention. Given the modest net benefit and limited threshold range, these results require confirmation in larger external cohorts. The model may assist clinicians in identifying patients who could benefit from enhanced surveillance, individualized preventive measures, or closer follow up during chemotherapy, but it should not be used as a definitive treatment guide.

Several limitations must be acknowledged. First, the limited sample size and event number represent the primary constraint of this prediction model. Among 246 included patients, only 55 pulmonary infection events occurred, yielding an overall EPV of approximately 5.5. In the training cohort, 47 events among 196 patients corresponded to an EPV of approximately 4.7, which falls below conventional recommendations and may increase the risk of overfitting and coefficient instability (27). To mitigate this concern, we restricted candidate predictors to clinically relevant variables, applied Forward-AIC based selection rather than simple P-value driven approaches, and performed bootstrap validation with repeated variable selection to estimate optimism and assess predictor stability. Nevertheless, residual uncertainty about model robustness remains.

Second, the relatively low infection incidence (22.4%) resulted in an imbalanced outcome distribution, which may affect threshold dependent classification metrics, particularly precision estimates. Although we deliberately avoided oversampling techniques such as SMOTE to preserve the original probability structure and calibration properties, future studies with larger event numbers and more balanced cohorts are needed to further validate model performance.

Third, this was a single center retrospective study, and the validation cohort was small (n=50, with 8 events), limiting the precision of calibration assessment. External validation using geographically and clinically diverse cohorts was not available; therefore, the generalizability and transportability of the nomogram require further investigation. Although multiple imputation sensitivity analyses yielded results consistent with the complete case analysis, the missing at random assumption cannot be fully verified in retrospective data.

Finally, the current nomogram was developed using static pre-chemotherapy variables and does not incorporate longitudinal changes during treatment. Although exploratory landmark analysis suggested that baseline predictors maintained acceptable discrimination across chemotherapy cycles, dynamic information such as cycle specific neutrophil decline, inflammatory marker fluctuations, nutritional deterioration, and treatment related complications were not captured. Future prospective multicenter studies should focus on external validation, model recalibration, and the development of longitudinal dynamic prediction models that incorporate time updated clinical and laboratory parameters to enable individualized risk reassessment throughout the course of chemotherapy.

## 5 Conclusions

We developed and internally validated a nomogram for predicting pulmonary infection risk during chemotherapy in patients with NSCLC. The model uses five routine pre-chemotherapy indicators: ANC, serum albumin, age, concurrent immunotherapy, and COPD. After optimism correction, the model maintained moderate discriminative ability (AUC 0.735) and effectively stratified patients into three distinct risk levels, which may aid clinicians in identifying high risk individuals before treatment initiation and guiding stratified surveillance protocols. Owing to the single center retrospective design, modest sample size, low EPV, and absence of independent external validation, the current nomogram should be considered exploratory rather than definitive. Larger multicenter prospective studies are required to externally validate, recalibrate, and potentially update this model before routine clinical implementation.

## Data Availability

The original contributions presented in the study are included in the article/**Supplementary Material**. Further inquiries can be directed to the corresponding author.

## Supplementary material

Supplementary Table 1 Final logistic regression equation and model coefficients.
