# Supplementary material for: Prediction of pulmonary infection during chemotherapy in non-small cell lung cancer: development and internal validation of a nomogram
Source: Front Oncol. 2026 Sep 4;16:1926061. doi: 10.3389/fonc.2026.1926061 (PMC13585531; doi:10.3389/fonc.2026.1926061)
Supplement: Supplementary file 1 [file Table1.doc]

Supplementary Table S1. Final logistic regression equation and model coefficients

| **Predictor** | **Coding** | **β coefficient** | **SE** | **OR (95% CI)** |
| --- | --- | --- | --- | --- |
| Intercept | — | 1.842 | 0.865 | — |
| ANC (×10⁹/L) | continuous | -0.544 | 0.142 | 0.58 (0.44–0.77) |
| Albumin (g/L) | continuous | -0.083 | 0.039 | 0.92 (0.85–0.99) |
| Age (year) | continuous | 0.049 | 0.02 | 1.05 (1.01–1.10) |
| Immunotherapy | 0=no,1=yes | 0.881 | 0.387 | 2.41 (1.13–5.15) |
| COPD | 0=no,1=yes | 0.724 | 0.414 | 2.06 (0.92–4.62) |
